# A nomogram based on platelet-to-lymphocyte ratio for predicting pathological complete response of breast cancer after neoadjuvant chemotherapy

**DOI:** 10.1186/s12885-023-10703-x

**Published:** 2023-03-14

**Authors:** Rulan Ma, Wanzhen Wei, Haixia Ye, Chengxue Dang, Kang Li, Dawei Yuan

**Affiliations:** 1 Department of Surgical Oncology, The First Affiliated Hospital of Xi’an Jiaotong University, Shannxi, 710061 Xi’an China; 2 Department of Hepatobiliary Surgery, The First Affiliated Hospital of Xi’an Jiaotong University, Shannxi, 710061 Xi’an China; 3 The Second Clinical College, Department of Medicine, Wuhan University, Hubei, 430071 Wuhan China

**Keywords:** Breast cancer, Neoadjuvant chemotherapy, Pathological complete response, Nomogram, Prediction model

## Abstract

**Objective:**

To investigate the role of platelet-to-lymphocyte ratio (PLR) in complete pathological response (pCR) of breast cancer (BC) patients after neoadjuvant chemotherapy (NAC), as well as to establish and validate a nomogram for predicting pCR.

**Methods:**

BC patients diagnosed and treated in the First Affiliated Hospital of Xi’an Jiaotong University from January 2019 to June 2022 were included. The correlation between pCR and clinicopathological characteristics was analyzed by Chi-square test. Logistic regression analysis was performed to evaluate the factors that might affect pCR. Based on the results of regression analysis, a nomogram for predicting pCR was established and validated.

**Results:**

A total of 112 BC patients were included in this study. 50.89% of the patients acquired pCR after NAC. Chi-square test showed that PLR was significantly correlated with pCR (X^2^ = 18.878, P < 0.001). And the PLR before NAC in pCR group was lower than that in Non-pCR group (t = 3.290, P = 0.001). Logistic regression analysis suggested that white blood cell (WBC) [odds ratio (OR): 0.19, 95% confidence interval (CI): 0.04–0.85, P = 0.030)], platelet (PLT) (OR: 0.19, 95%CI: 0.04–0.85, P = 0.030), PLR (OR: 0.18, 95%CI: 0.04–0.90, P = 0.036) and tumor grade (OR: 9.24, 95%CI: 1.89–45.07, P = 0.006) were independent predictors of pCR after NAC. A nomogram prediction model based on WBC, PLR, PLR and tumor grade showed a good predictive ability.

**Conclusion:**

PLR, PLT, WBC and tumor grade were independent predictors of pCR in BC patients after NAC. The nomogram based on the above positive factors showed a good predictive ability.

## Introduction

Breast cancer (BC) is the most common malignant tumor in the world, accounting for about 11.7% of new cancer cases[1]. In the past few decades, researchers have conducted a series of clinical trials to establish a standard treatment for BC[2–4]. It was reported that neoadjuvant chemotherapy (NAC) can significantly prolong the overall survival of the patients with BC, which makes NAC a recommended treatment for advanced BC[5, 6]. Previous studies have shown that pathological complete response (pCR) in patients treated with NAC is significantly related to the clinical prognosis [7, 8]. Therefore, it is necessary to identify the factors that may affect pCR of BC patients after NAC and establish related prediction models.

Previous studies have shown that the host immune system plays an important role in the occurrence, development and metastasis of tumor[9, 10]. Peripheral blood inflammatory indexes, including neutrophil-to-lymphocyte ratio (NLR), platelet-to-lymphocyte (PLR), lymphocyte-to-monocyte ratio (LMR) and systemic immune inflammation index (SII), were significantly correlated with the prognosis of BC patients[11–13]. For example, high NLR and PLR were associated with shorter overall survival and disease-free survival, while low LMR was associated with improved prognosis[12, 14]. Besides, the elevated PLR was related to the risk of lymph node metastasis and distant metastasis[13]. In addition, PLR was an independent factor for predicting pCR after NAC for BC, and patients with low PLR achieved higher pCR rates[15–17]. However, another study showed that patients with high PLR had higher pCR rates than those with low PLR[18]. It was also reported that there was no significant correlation between PLR and pCR[19]. Therefore, the role of PLR in pCR of BC after NAC is still controversial. Moreover, there is still a lack of PLR-based nomogram for predicting pCR in BC patients receiving NAC.

Therefore, this study aimed to determine the role of PLR in pCR after NAC for BC patients, as well as to establish and validate the nomogram prediction model based on PLR for predicting pCR of BC patients receiving NAC.

## Methods

### Study population

A total of 112 BC patients from the First Affiliated Hospital of Xi’an Jiaotong University from January 2019 to June 2022 were included in this study. The inclusion criteria are as follows: (1) preoperative pathological diagnosis indicated BC; (2) received neoadjuvant chemotherapy; (3) surgery after NAC was performed; (4) clinicopathological data and postoperative pathological data were completed. The exclusion criteria are as follows: (1) received other anti-tumor therapy before NAC; (2) withdrawal from NAC; (3) surgery after NAC was not performed; (4) clinicopathological data was incomplete. This study was performed in accordance with the Declaration of Helsinki, and was carried out under the approval and supervision of the Ethics Committee of the First Affiliated Hospital of Xi’an Jiaotong University (No. XJTU1AF2022LSK-335). The study was a retrospective study, and written informed consent for participation was not required for this study in accordance with the national legislation and the institutional requirements. Therefore, the waiver of informed consent was approved by the Ethics Committee of the First Affiliated Hospital of Xi’an Jiaotong University.

### Data collection and processing

The baseline data, clinicopathological data, treatment-related data and laboratory examination data before NAC were collected. The data were processed by Microsoft Excel and SPSS26.0 softwares. The optimal cut-off value of continuous data was calculated via the receiver operating characteristic (ROC) curve. Then, the continuous variables were converted into binary variables according to the cut-off values.

### Statistical analysis

SPSS26.0 and RStudio softwares were used for statistical analysis. The difference between the two groups was analyzed by Chi-square test or student t-test. Univariate and multivariate Logistic regression analyses were used to identify the factors that might be related to pCR after NAC. According to the results of multivariate Logistic regression analysis, a nomogram prediction model of pCR after NAC was established. The concordance index (C-index), ROC curve, Bootstrap calibration curve, decision curve analysis (DCA) and clinical impact curve (CIC) were used to validate the predictive ability of the nomogram prediction model. P < 0.05 was considered to be statistically significant.

## Results

### Baseline data of BC patients received NAC

**Fig. 1:**
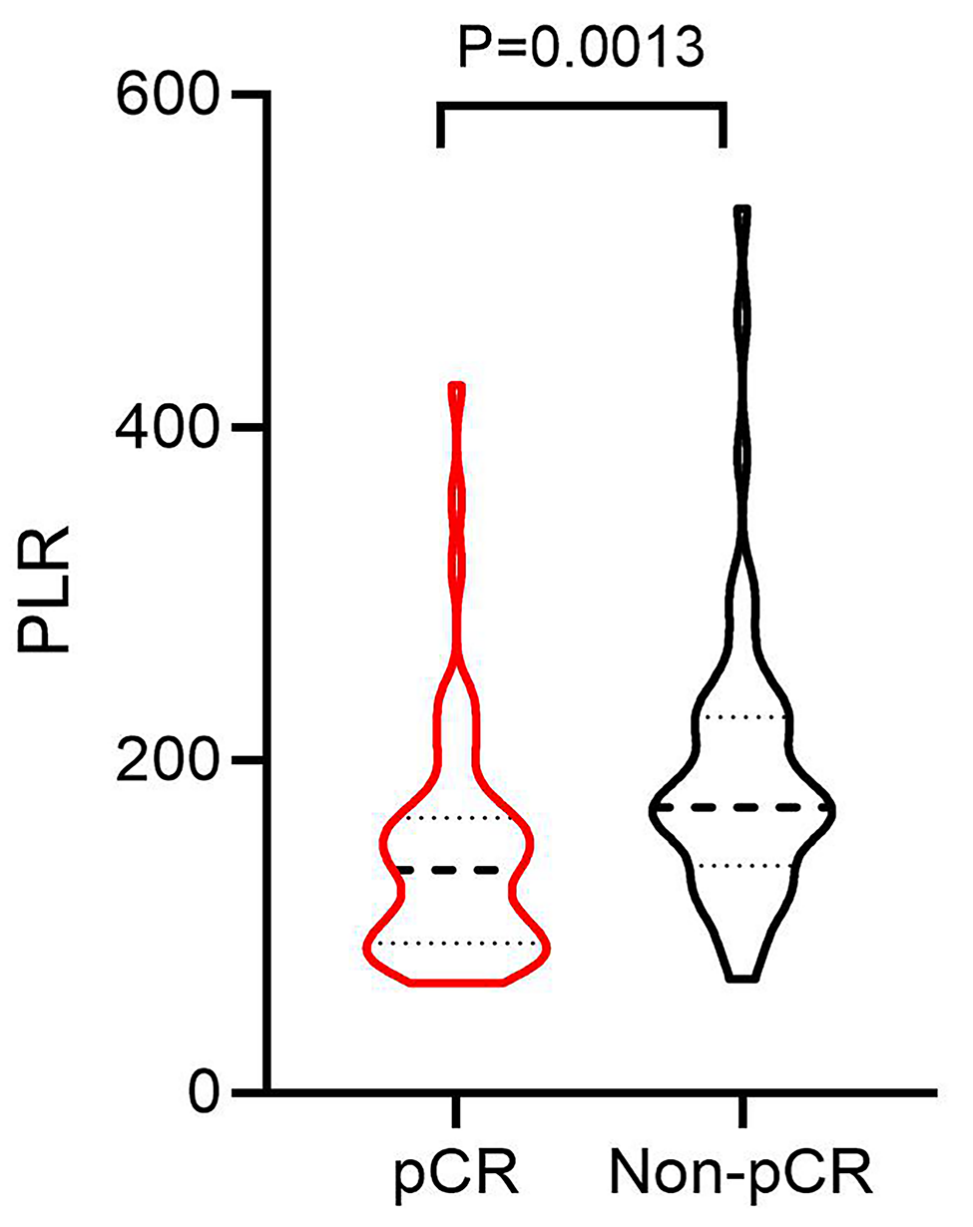
**The difference of PLR before NAC in pCR and Non-pCR patients with breast cancer.** PLR: Platelet-to-lymphocyte ratio; NAC: Neoadjuvant chemotherapy; pCR: Pathological complete response

A total of 112 BC patients were included in this study. The average age of the patients was 50.94 ± 8.43 years old. All patients received preoperative NAC and operation. Postoperative pathology confirmed that 50.89% (57/112) of the patients acquired pCR. The clinicopathological characteristics of BC patients were shown in Table 1. Chi-square test showed that tumor grade, estrogen receptor (ER), molecular classification, neoadjuvant therapy cycle, white blood cell (WBC), platelet (PLT), lymphocyte (LYM), NLR, PLR, LMR and SII were significantly correlated with pCR of BC patients after NAC. Of note, PLR in pCR group and Non-pCR group was significantly different. The PLR before NAC in pCR group was lower than that in Non-pCR group (t = 3.290, P = 0.001) (Fig. 1).

**Table 1:** Baseline data of BC patients receiving NAC

Factors		Non-pCR (N = 55)	pCR (N = 57)	Total	X^2^	P
**Age (Years)**	<44.5	9	16	25	2.212	0.137
	≥ 44.5	46	41	87		
**Menarche age (Years)**	<13.5	27	35	62	1.717	0.190
	≥ 13.5	28	22	50		
**Menstrual states**	No	13	15	28	0.107	0.743
	Yes	42	42	84		
**Menopausal age (Years)**	<51.5	37	30	67	3.614	0.057
	≥ 51.5	5	12	17		
**Primiparous age (Years)**	<22.5	24	32	56	1.751	0.186
	≥ 22.5	31	25	56		
**Number of births**	<3	43	50	93	1.808	0.179
	≥ 3	12	7	19		
**cT**	1/2	45	47	92	0.008	0.930
	3/4	10	10	20		
**cN**	0/1	37	35	72	0.517	0.472
	2/3	14	18	32		
**cM**	0	50	52	102		1.000^a^
	1	2	3	5		
**Axillary lymph nodes**	Negative	11	6	17	1.951	0.162
	Posotive	14	51	65		
**Pathological type**	IDC	55	53	108		0.119^a^
	Other	0	4	4		
**Grade**	1/2	47	34	81	4.764	**0.029**
	3	7	15	22		
**ER**	Negative	16	28	44	4.404	**0.036**
	Positive	30	29	59		
**PR**	Negative	19	27	46	1.696	0.193
	Positive	35	30	65		
**HER-2**	Negative	13	8	21	1.822	0.177
	Positive	41	49	90		
**Ki-67**	<42.50	36	28	64	3.049	0.081
	≥ 42.50	19	29	48		
**Molecular classification**	Luminal A	0	1	1		0.063^a^
	Luminal B(HER-2^−^)	10	3	13		
	Luminal B(HER-2^+^)	28	25	53		
	HER-2^+^	13	24	37		
	Triple negative	3	4	7		
**Therapy cycle**	<7	28	18	46	4.321	**0.038**
	≥ 7	27	39	66		
**CEA (ng/mL)**	<2.93	42	35	77	3.682	0.055
	≥ 2.93	11	21	32		
**CA153 (U/mL)**	<19.14	35	31	66	1.300	0.254
	≥ 19.14	18	35	53		
**Hb (g/L)**	<135.50	30	35	65	0.541	0.462
	≥ 135.50	25	22	47		
**WBC (×10^9/L)**	<6.13	13	23	36	4.475	**0.034**
	≥ 6.13	43	34	77		
**PLT (×10^9/L)**	<247.50	27	17	44	4.356	**0.037**
	≥ 247.50	28	40	68		
**LYM (×10^9/L)**	<1.64	35	13	48	0.352	**<0.001**
	≥ 1.64	20	44	64		
**NEU (×10^9/L)**	<4.70	25	33	58	1.735	0.188
	≥ 4.70	30	24	54		
**Monocyte (×10^9/L)**	<0.44	38	47	85	2.733	0.098
	≥ 0.44	17	10	27		
**ALB (g/L)**	<52.15	5	2	7		0.434^a^
	≥ 52.15	41	43	84		
**NLR**	<2.02	6	28	34	19.334	**<0.001**
	≥ 2.02	49	29	78		
**PLR**	<161.50	18	42	60	18.878	**<0.001**
	≥ 161.50	37	15	52		
**LMR**	<6.39	45	29	74	11.954	**0.001**
	≥ 6.39	10	28	38		
**SII**	<598.50	13	32	45	12.304	**<0.001**
	≥ 598.50	42	25	67		

^a^ Fisher exact probability method. BC: Breast cancer; NAC: Neoadjuvant chemotherapy; pCR: Pathological complete response; IDC: Invasive ductal carcinoma; ER: Estrogen receptor; PR: Progesterone receptor; HER-2: Human epidermal growth factor receptor-2; CEA: Carcinoembryonic antigen; CA153: Carbohydrate antigen 153: WBC: White blood cell; Hb: Hemoglobin; PLT: Platelet; LYM: Lymphocyte; ALB: Albumin; NLR: Neutrophil-to-lymphocyte ratio; PLR: Platelet-to-lymphocyte ratio; LMR: Lymphocyte-to-monocyte ratio; SII: Systemic immune inflammation index.

### Logistic regression analysis for detecting the factors related to pCR after NAC

In order to identify the factors that might affect the pCR of BC patients after NAC, we performed univariate Logistic regression analysis. The results showed that pCR was significantly correlated with tumor grade (P = 0.033), ER (P = 0.037), molecular classification (P = 0.046), therapy cycle (P = 0.039), WBC (P = 0.037), PLT (P = 0.038), LYM (P < 0.001), NLR (P < 0.001), PLR (P < 0.001), LMR (P = 0.001) and SII (P = 0.001) (Table 2). Further multivariate analysis showed that WBC [OR: 0.19, 95% confidence interval (CI):0.04–0.85, P = 0.030], PLT (OR:10.62, 95%CI: 2.24–50.30, P = 0.003), PLR (OR:0.18, 95%CI: 0.04–0.90, P = 0.036) and tumor grade (OR:9.24, 95%CI: 1.89–45.07, P = 0.006) were independent predictors of pCR in BC patients after NAC (Table 3). Among them, PLR and WBC were negatively correlated with pCR, while PLT and tumor grade were positively correlated with pCR.

**Table 2:** Univariate Logistic analysis for pCR of BC cancer after NAC

Factors		Univariate analysis			
		OR	95%CI		P
			Lower	Upper	
**Age (Years)**	≥ 44.5 vs<44.5	0.50	0.20	1.26	0.141
**Menarche age (Years)**	≥ 13.5 vs<13.5	0.61	0.29	1.28	0.191
**Menstrual states**	Menopausal vs. Premenopausal	0.87	0.37	2.04	0.743
**Menopausal age (Years)**	≥ 51.5 vs<51.5	2.96	0.94	9.34	0.064
**Primiparous age (Years)**	≥ 22.5 vs<22.5	0.60	0.29	1.28	0.187
**Number of births**	≥ 3 vs<3	0.50	0.18	1.39	0.184
**cT**	3/4 vs. 1/2	0.96	0.36	2.52	0.930
**cN**	2/3 vs. 0/1	1.36	0.59	3.14	0.473
**cM**	1 vs. 0	1.44	0.23	9.00	0.695
**Axillary lymph nodes**	Positive vs. Negative	2.12	0.73	6.22	0.169
**Pathological type**	Other vs. IDC	1.68E + 09	0.00		0.999
**Grade**	3 VS 1/2	2.96	1.09	8.05	**0.033**
**ER**	Positive vs. Negative	0.44	0.20	0.95	**0.037**
**PR**	Positive vs. Negative	0.60	0.28	1.29	0.194
**HER-2**	Positive vs. Negative	1.94	0.73	5.14	0.181
**Ki-67**	≥ 42.50 vs<42.50	1.96	0.92	4.20	0.082
**Molecular classification**	Triple negative vs. HER-2^+^ vs. Luminal B(HER-2^+^) vs. Luminal B(HER-2^+^) vs. Luminal A	1.65	1.01	2.71	**0.046**
**Therapy cycle**	≥ 7 vs<7	2.25	1.04	4.85	**0.039**
**CEA (ng/mL)**	≥ 2.93 vs<2.93	2.29	0.97	5.39	0.058
**CA153(U/mL)**	≥ 19.14 vs<19.14	1.57	0.72	3.40	0.255
**Hb**	≥ 135.50 vs<135.50	0.75	0.36	1.60	0.463
**WBC**	≥ 6.13 vs<6.13	0.41	0.18	0.95	**0.037**
**PLT (×10^9/L)**	≥ 247.50 vs<247.50	2.27	1.04	4.93	**0.038**
**LYM**	≥ 1.64 vs<1.64	5.92	2.59	13.55	**<0.001**
**NEU (×10^9/L)**	≥ 4.70 vs<4.70	0.61	0.29	1.28	0.189
**Monocyte (×10^9/L)**	≥ 0.44 vs<0.44	0.48	0.20	1.16	0.102
**ALB (g/L)**	≥ 52.15 vs<52.15	2.62	0.48	14.28	0.265
**NLR**	≥ 2.02 vs. <2.02	0.13	0.05	0.34	**<0.001**
**PLR**	≥ 161.50 vs<161.50	0.17	0.08	0.39	**<0.001**
**LMR**	≥ 6.39 vs<6.39	4.34	1.84	10.26	**0.001**
**SII**	≥ 598.50 vs<598.50	0.24	0.11	0.55	**0.001**

BC: Breast cancer; NAC: Neoadjuvant chemotherapy; pCR: Pathological complete response;

OR: Odds ratio; CI: Confidence interval; IDC: Invasive ductal carcinoma; ER: Estrogen receptor; PR: Progesterone receptor; HER-2: Human epidermal growth factor receptor-2; CEA: Carcinoembryonic antigen; CA153: Carbohydrate antigen 153: WBC: White blood cell; Hb: Hemoglobin; PLT: Platelet; LYM: Lymphocyte; ALB: Albumin; NLR: Neutrophil-to-lymphocyte ratio; PLR: Platelet-to-lymphocyte ratio; LMR: Lymphocyte-to-monocyte ratio; SII: Systemic immune inflammation index.

**Table 3:** Multivariate Logistic analysis for pCR of BC cancer after NAC

Factors		Multivariate analysis			
		OR	95%CI		P
			Lower	Upper	
**WBC (×10** ^**9**^ **/L)**	≥ 6.13 vs. <6.13	0.19	0.04	0.85	**0.030**
**PLT (×10** ^**9**^ **/L)**	≥ 247.50 vs. <247.50	10.62	2.24	50.30	**0.003**
**PLR**	≥ 161.50 vs. <161.50	0.18	0.04	0.90	**0.036**
**Grade**	3 vs. 1/2	9.24	1.89	45.07	**0.006**

BC: Breast cancer; NAC: Neoadjuvant chemotherapy; pCR: Pathological complete response;

OR: Odds ratio; CI: Confidence interval; WBC: White blood cell; PLT: Platelet; PLR: Platelet-to-lymphocyte ratio.

### Establishment and validation of the nomogram for predicting pCR of BC patients after NAC

**Fig. 2:**
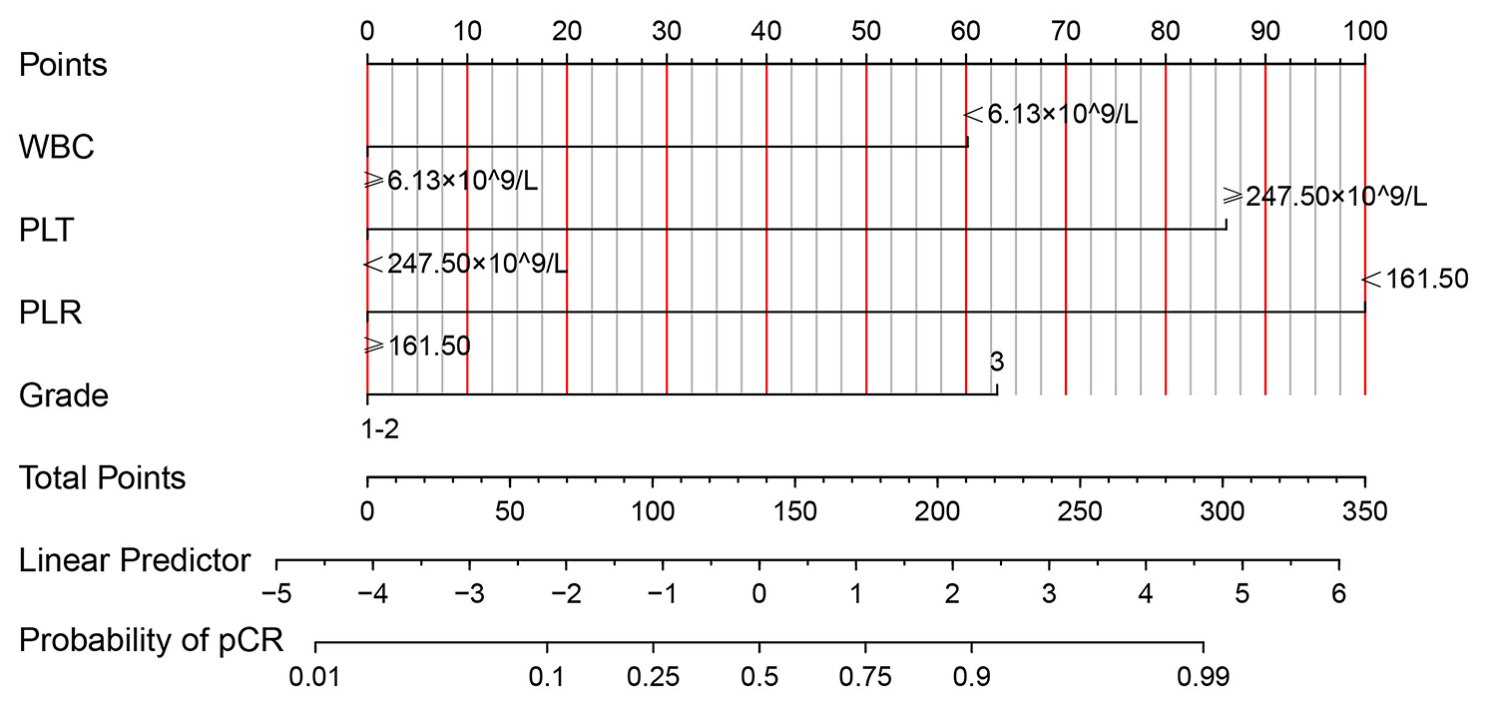
** A nomogram for predicting pCR in breast cancer after NAC.** The individual point represents the corresponding point of each factor, and the total point represents the sum of individual point of all factors. Probability corresponding to the total point is the probability of pCR in breast cancer patients after NAC. pCR: Pathological complete response; NAC: Neoadjuvant chemotherapy; WBC: White blood cell; PLT: Platelet; PLR: Platelet-to-lymphocyte ratio

**Fig. 3:**
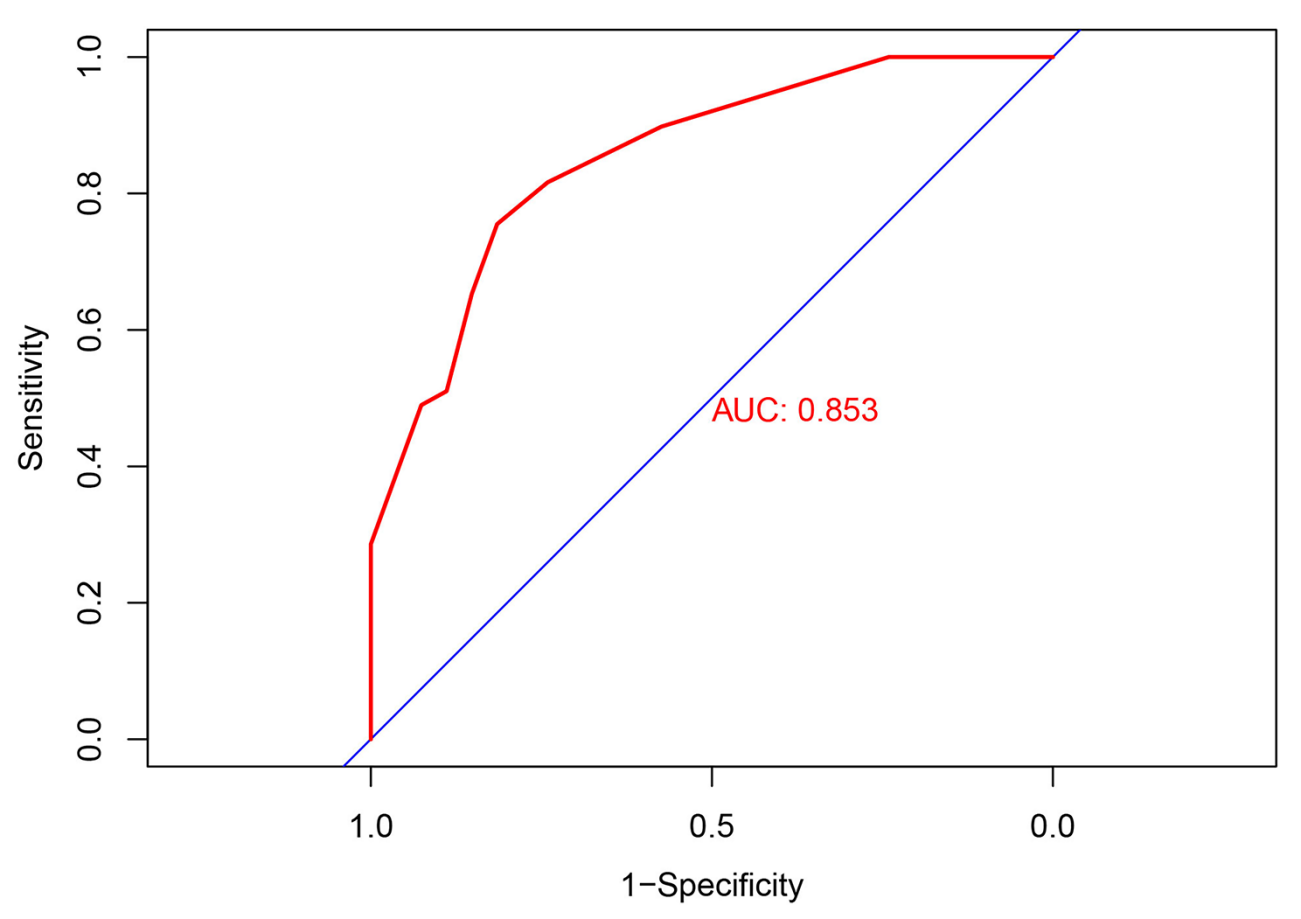
**Receiver operating characteristic curve of the nomogram for predicting pCR in breast cancer patients treated with NAC.** The closer the AUC of ROC curve is to 1, the better the ability of the nomogram to predict pCR after NAC. pCR: Pathological complete response; NAC: Neoadjuvant chemotherapy; AUC: Area under the curve

**Fig. 4:**
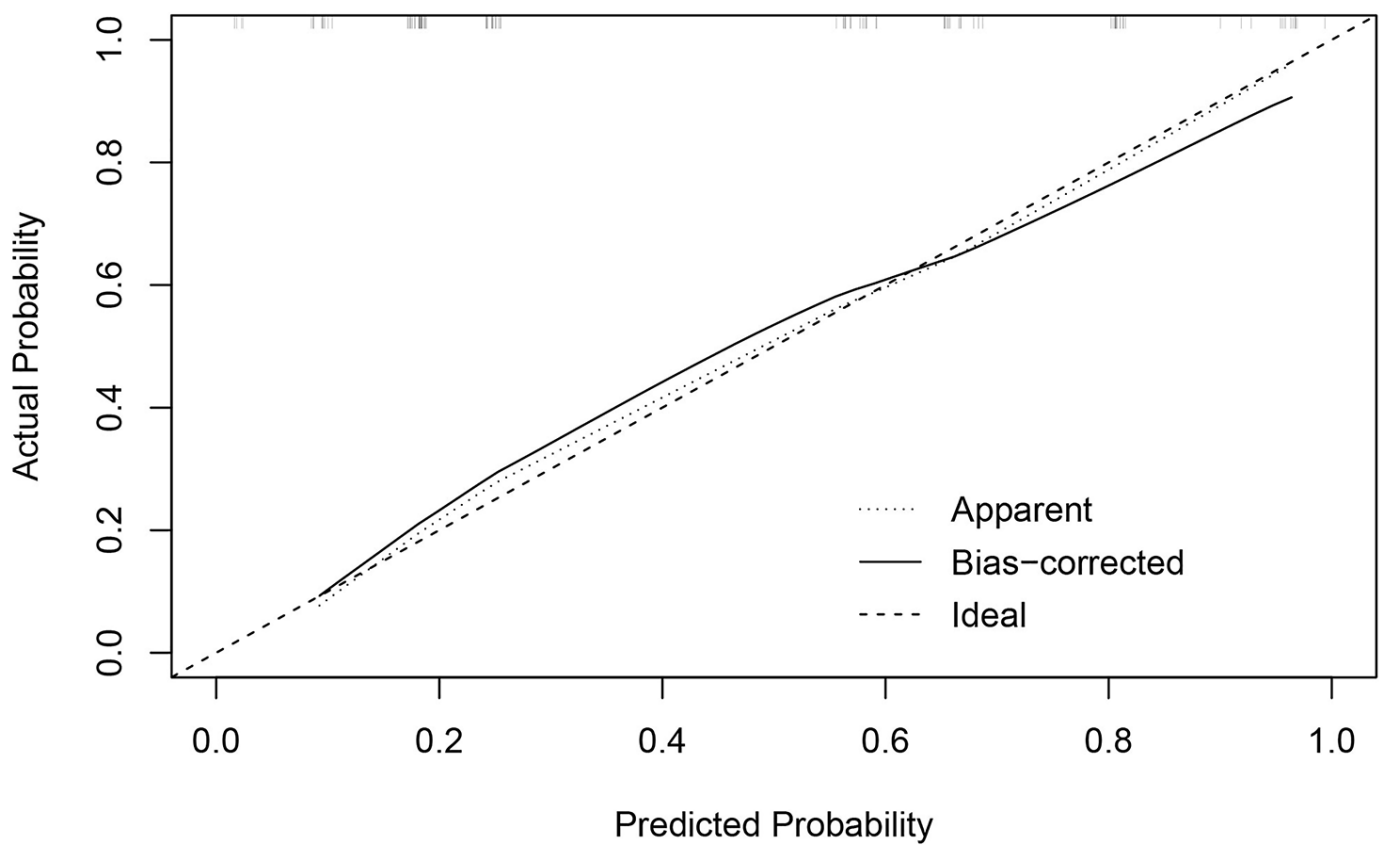
**Bootstrap calibration curve of the nomogram for predicting pCR in breast cancer received NAC.** The X-axis represents the predicted probability, and the Y-axis represents the actual probability. The validation curve shows that the trend of the predicted value is consistent with the true value, and there is a good calibration effect between the predicted value and actual observed value, indicating predictive ability of the nomogram as good. pCR: Pathological complete response; NAC: Neoadjuvant chemotherapy

**Fig. 5:**
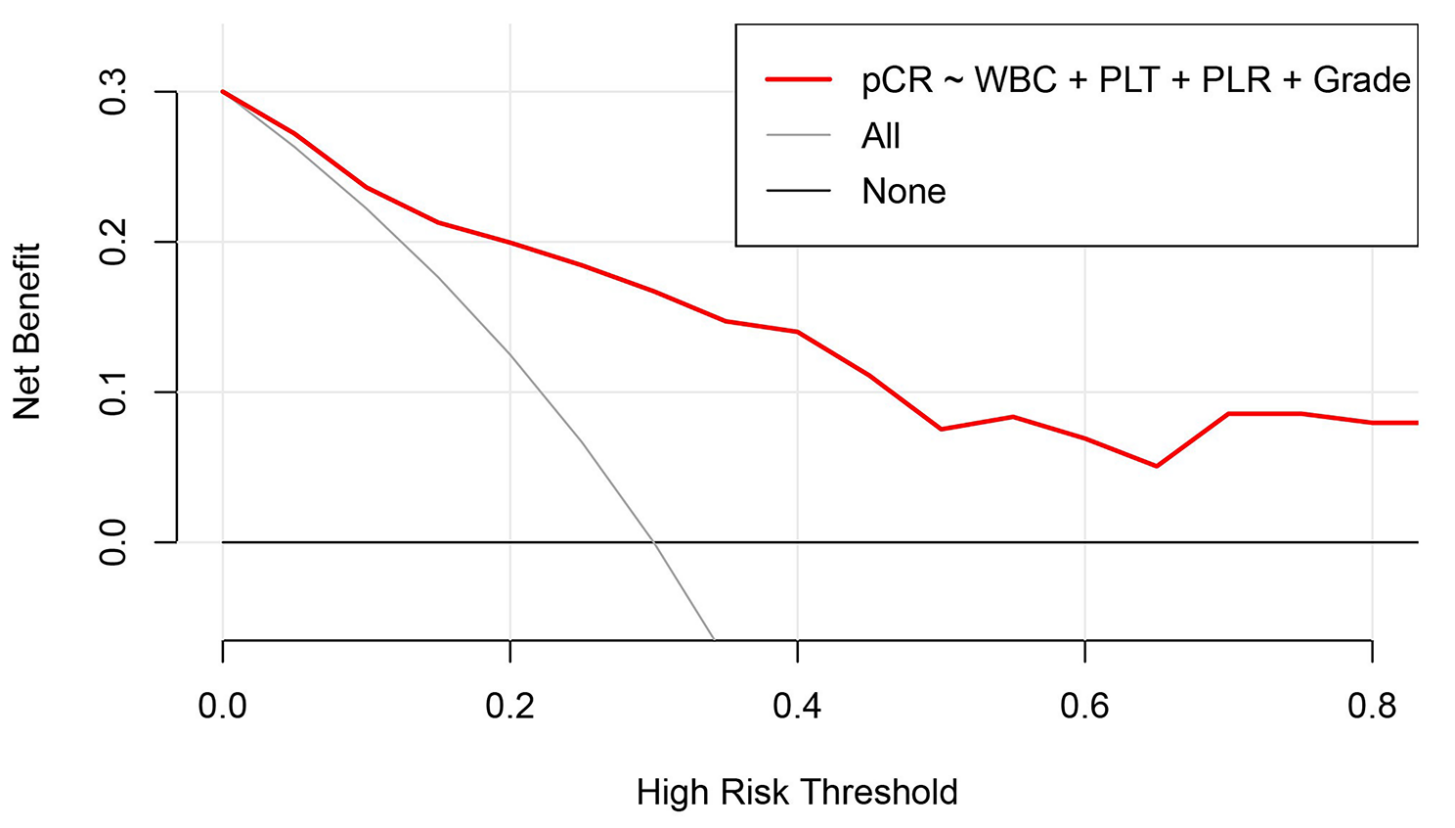
**Decision curve analysis of the nomogram for predicting pCR in breast cancer after NAC.** The X-axis represents the risk threshold probability, and the Y-axis represents the net benefit. The decision curve analysis shows that the nomogram has a higher net benefit than the default treat-all and treat-none, indicating that the probability of pCR predicted by the nomogram is superior to either treat-all or none strategy. pCR: Pathological complete response; NAC: Neoadjuvant chemotherapy; WBC: White blood cell; PLT: Platelet; PLR: Platelet-to-lymphocyte ratio

**Fig. 6:**
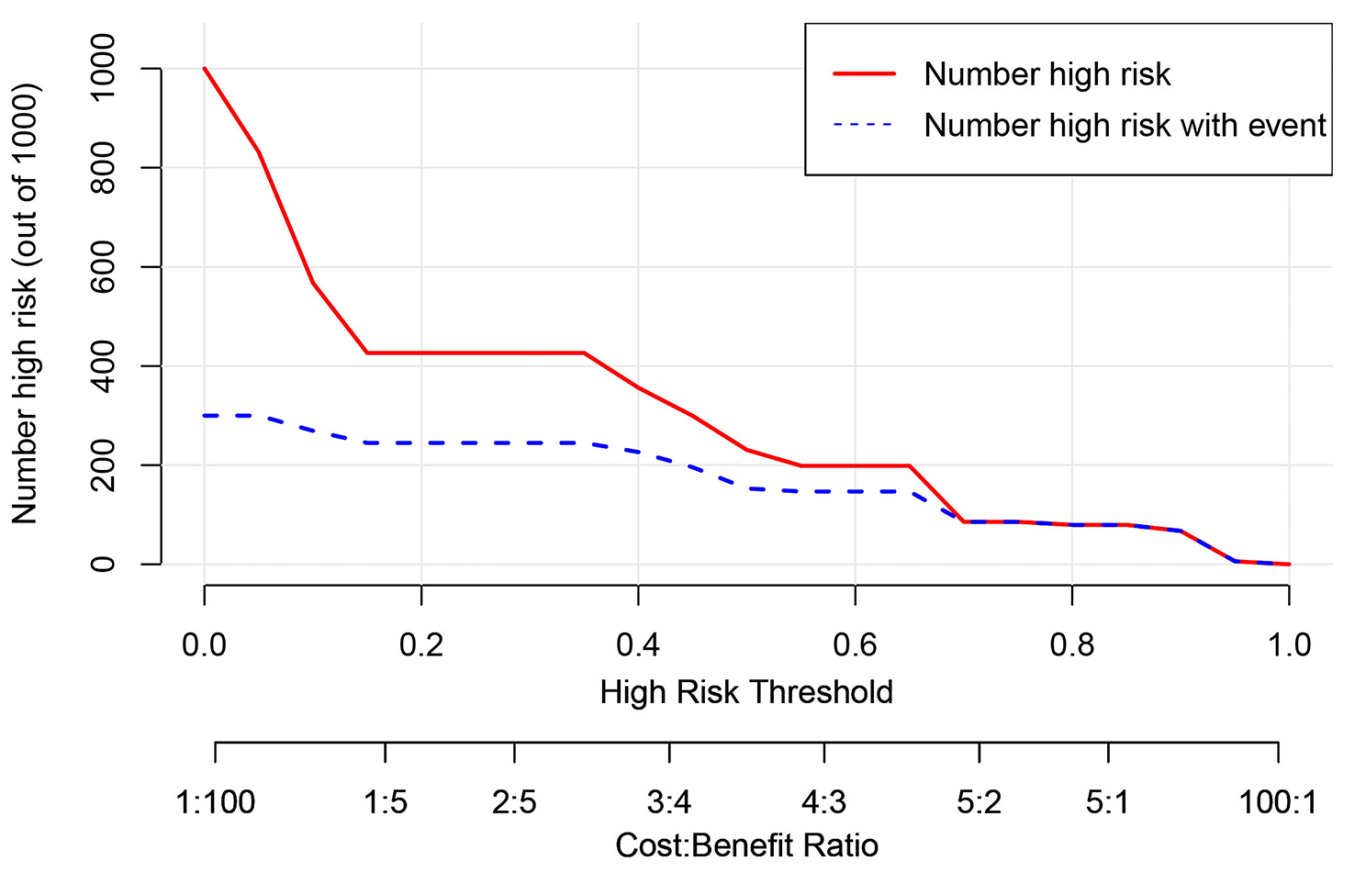
**Clinical impact curve of the nomogram for predicting pCR in breast cancer received NAC.** The red curve shows the predicted improved number at different threshold probabilities and the blue curve represents actual improved patients. The predictive improved number is close to the actual number of positive cases when the threshold probability was greater than 0.2. At this time, the Cost: Benefit ratio was 0.2. It is suggested that the nomogram could accurately predict the probability of pCR after NAC. pCR: Pathological complete response; NAC: Neoadjuvant chemotherapy

Based on the result of multivariate Logistic regression analysis, a nomogram was established to predict pCR after NAC (Fig. 2). The C-index of the prediction model was 0.853 [95% confidence interval (CI): 0.782–0.924], and the area under the curve (AUC) shown by the ROC curve was 0.853 (Fig. 3), indicating that the discrimination ability of the model was good. The Bootstrap calibration curve also showed that the nomogram had a good prediction ability (the average absolute error was 0.030) (Fig. 4). In addition, the DCA curve and CIC curve showed that the nomogram had accuracy to predict pCR of the patients who received NAC (Figs. 5 and 6).

## Discussion

In this study, the role of PLR in pCR in BC patients after NAC was investigated, and other possible factors affecting pCR were explored. Finally, according to the results of Logistic regression analysis, a nomogram prediction model based on PLR was established, and a variety of internal validation methods were used to verify the nomogram prediction model.

The application of NAC for BC has greatly improved the prognosis of patients, and the acquisition of pCR after NAC is also significantly related to the prolonged survival time of patients[6]. Previous studies have shown that PLR can be used as a predictor and prognostic factor of treatment response to NAC for BC. Low PLR was associated with prolonged overall survival and disease-free survival[12], while high PLR was associated with increased risk of lymph node and distant metastasis in BC patients[13]. PLR can also predict the pathological response of BC patients after NAC. For example, patients with low PLR were more likely to achieve pCR after NAC than patients with high PLR^[15–17]^. However, another study suggested that there was no significant correlation between the level of PLR and the rate of pCR[19]. Previous study even suggested that the higher the PLR value of patients before NAC, the greater the probability of acquiring pCR[18]. Our study showed that there was a significant difference in PLR value between the pCR group and the Non-pCR group, and the PLR was significantly lower in the pCR group. Through regression analysis, it was found that high PLR (> 161.50) was an independent predictor of pCR, which is consistent with the results of most previous studies^[15–17]^. Our results strongly confirmed that low PLR (≤ 161.50) was associated with a high pCR rate, indicating that PLR before NAC could be used as a feasible indicator for predicting pCR of BC patients.

The nomogram is a simple and effective tool for predicting the outcome[20]. The nomogram prediction model established based on the results of regression analysis can predict the pathological response of tumor patients after NAC[21–24], and the prediction ability of these models is pretty good. Multivariate regression analysis in our study showed that PLR, PLT, WBC and tumor grade were independent predictive factors of pCR. Based on the above positive indicators, we established a nomogram that could predict the pCR in BC patients receiving NAC. The C-index of the nomogram was 0.853 (95%CI: 0.782–0.924), and the AUC value of the ROC curve was 0.853, indicating that the nomogram prediction model had good accuracy in predicting pCR. We also validated the nomogram internally through the calibration curve, DCA curve and CIC curve, and the results showed that our prediction model had a good prediction ability. Therefore, this study provided a simple and feasible prediction model for BC patients with pCR after NAC.

But there are still some limitations in this study. First of all, the population included in this study came from a single center, and the sample size was not large enough. Secondly, this study was a retrospective analysis, so it was difficult to obtain more comprehensive clinical data, so only common clinicopathological factors and laboratory indicators were analyzed. Finally, due to the lack of external data, the nomogram model established in this study was only verified via internal validation. Therefore, in the next step, it is necessary to enlarge the sample size and analyze more indicators to improve the prediction model.

## Conclusion

This study confirmed the predictive role of PLR in pCR of BC patients following NAC. Based on Logistic regression analysis, it was found that WBC, PLT, PLR and tumor grade were independent predictors of pCR. The nomogram prediction model based on the above four positive indicators showed a good ability to predict pCR after NAC.

## Data Availability

All data generated or analyzed during this study are included in this published article.
